# The role of pyroptosis-related genes in the diagnosis and subclassification of sepsis

**DOI:** 10.1371/journal.pone.0293537

**Published:** 2023-11-08

**Authors:** Wencong Ding, Laping Huang, Yifeng Wu, Junwei Su, Liu He, Zhongxiang Tang, Min Zhang

**Affiliations:** 1 Department of Nephrology, Affiliated Guangdong Hospital of Integrated Traditional Chinese and Western Medicine of Guangzhou University of Chinese Medicine, Foshan, 528000, Guangdong, China; 2 Intensive Care Unit, Affiliated Guangdong Hospital of Integrated Traditional Chinese and Western Medicine of Guangzhou University of Chinese Medicine, Foshan, 528000, Guangdong, China; Renji Hospital, Shanghai Jiao Tong University School of Medicine, CHINA

## Abstract

Pyroptosis is a new form of programmed cell death recognized as crucial in developing sepsis. However, there is limited research on the mechanism of pyroptosis-related genes in sepsis-related from the Gene Expression Omnibus (GEO) database and standardized. The expression levels of pyroptosis-related genes were extracted, and differential expression analysis was conducted. A prediction model was constructed using random forest (RF), support vector machine (SVM), weighted gene co-expression new analysis (WGCNA), and nomogram techniques to assess the risk of sepsis. The relationship between pyroptosis-related subgroups and the immune microenvironment and inflammatory factors was studied using consistent clustering algorithms, principal component analysis (PCA), single-sample genomic enrichment analysis (ssGSEA), and immune infiltration. A risk prediction model based on 3 PRGs has been constructed and can effectively predict the risk of sepsis. Patients with sepsis can be divided into two completely different subtypes of pyroptosis-related clusters. Cluster B is highly correlated with the lower proportion of Th17 celld and has lower levels of expression of inflammatory factors. This study utilizes mechanical learning methods to further investigate the pathogenesis of sepsis, explore potential biomarkers, provide effective molecular targets for its diagnosis and treatment of sepsis.

## Introduction

Sepsis is a life-threatening multi-organ dysfunction syndrome that arises when the host exhibits a dysfunctional response to infection [1]. The incidence rate of sepsis is high, and the disease progresses rapidly, often leading to multiple-organ failure [2]. Without timely intervention, the prognosis for patients with sepsis is mostly discouraging. A meta-analysis comprising 51 studies revealed an incidence rate of 189 sepsis cases per 100000 person-years, with a mortality rate of 26.7%. In the ICU, the incidence rate was 58 per 100000 person-years, with a mortality rate of 41.9% [3]. The Quick Sequential Organ Failure Score (qSOFA) is employed for the fast and convenient identification of sepsis patients and prediction of adverse outcomes in infected patients [4]. While the qSOFA score has high specificity for early risk assessment, its sensitivity is poor, resulting in delayed initiating of appropriate management of sepsis patients [5]. Adequate clinical and laboratory indicators to distinguish sepsis are required. Thus, highly specific and sensitive indicators are needed for early identification, intervention, and improved prognosis of patients with sepsis.

The mechanism involved in pyroptosis includes the activation of cysteine aspartate specific protease (Caspases) and a pro-inflammatory form of cell death mediated by gasdermin D (GSDMD) [6]. The characteristics of pyroptosis encompass the formation of micropores in the cell membrane, cell swelling, rupture, release of cell contents, secretion of inflammatory factors, promotion of innate immunity, and occurrence of cell death [7]. In response to injury, activation of either caspase-1 or caspase-11 leads to the cleavage of GSDMDresulting in the formation of an N-terminal fragment (GSDMD-N) that activates GSDMD. GSDMD-N localizes and aggregates into membrane pores on the cell membrane, inducing the production and release of inflammatory substances through these pores. Simultaneously, the accumulation of inflammatory substances within cells further activates NLRP3 inflammasomes, thereby amplifying the release of caspase-1-dependent inflammatory substances [8,9].

Several studies have demonstrated the significant role of inflammasome activation and dependent pyroptosis in the occurrence and development of sepsis [10–12]. Pyroptosis facilitates the release of pathogens by destroying infected cells and enabling their engulfment and elimination by immune cells, consequently reducing the body with antigens and eliminating intracellular pathogens. In sepsis, moderate pyroptosis is a protective mechanism against pathogenic microorganism infection, while over-activation of pyroptosis can exacerbate sepsis and septic shock [13,14]. Moreover, excessive activation of pyroptosis can result in organ damage, and the inhibition of pyroptosis could be a novel therapeutic approach for treating sepsis [15]. It should be emphasized that patients with sepsis need early diagnosis and treatment to prevent any delays in receiving optimal treatment. Machine learning and prediction models have demonstrated effectiveness in addressing this issue. Multiple prediction models have been developed and utilized to accurately predict the occurrence of various diseases [16,17]. In addition, the roles of pyroptosis in diagnosing and classifying sepsis have yet to be reported.

This study aimed to estimate the expression levels of pyroptosis-related genes (PRGs) in patients with sepsis and healthy controls and the correlation between PRGs. Multiple mechanical learning methods were applied to predict risk scores and identify key gene modules in patients with sepsis. The consensus clustering algorithm revealed two distinct subtypes of pyroptosis-related clusters. Furthermore, enrichment and immune infiltration analyses were conducted to investigate potential biomarkers and provide effective molecular targets for diagnosing and treating sepsis.

## Methods

### Data collection and processing

The GSE65682 dataset was obtained from the Gene Expression Omnibus (GEO) with the on-chip sequencing platform GPL13667. The dataset comprised blood samples from 760 sepsis patients and 42 healthy individuals.

### Analysis of differentially expressed genes (DEGs)

Based on published research, PRGs were identified [18,19]. Using the limma package in R software, the DEGs related to pyroptosis between sepsis patients and healthy controls were analyzed, setting the thresholds at |log2FC|>1 and Padj<0.05 [17]. Pearson correlation analysis was performed to reveal the associations between PRGs.

### Construction of protein-protein interaction (PPI) network

The STRING database was utilized to construct a protein-protein interaction (PPI) network for the DEGs; the species were set as Homo sapiens, retaining other settings as default.

### Construction of random forest (RF) and support vector machine (SVM) model

The random forest package in R software was used to construct an RF model. The PRGs were selected as independent variables, while patients with sepsis were considered dependent variables to predict the occurrence of sepsis disease. The SVM model is a widely used machine learning technique based on the structural risk minimization principle in statistical learning theory. The machine learning algorithm under supervision was employed to minimize classification errors and construct an SVM model. A comprehensive evaluation of the model was conducted by drawing a residual reverse cumulative distribution diagram and residual boxplot [20].

### Construction and validation of the nomogram

To predict the prevalence of sepsis, a nomograph was constructed based on the selection of the RF model from the previous step, using the rms package to select candidate PRGs [21]. The value of each level of influencing factor was assigned a score based on the outcome variable in the nomograph model. The total score was calculated by adding the individual score and the functional conversion relationship between the total score and the probability of the outcome event. The accuracy of the model was verified through a calibration curve assessing the consistency between the predicted and actual values.

### Construction of weighted gene co-expression network analysis (WGCNA)

To construct a gene co-expression network using the WGCNA package, the appropriate weighting coefficient is confirmed, which enables the generated co-expression network to have scale-free characteristics. The correlation matrix was converted into a topological overlap matrix using weighting coefficients, and the degree of dissimilarity was calculated. The WGCNA algorithm evaluates the correlation between module genes and disease grouping phenotypes, and the strength of the correlation was depicted in thermograms. A single module was considered significant when P<0.05. The module with the highest correlation coefficient with sepsis was selected as the key module. The Pearson correlation coefficient was calculated between each co-expression module and the characteristic gene values. Genes with module membership (MM)greater than 0.8 and gene significance (GS) greater than 0.65 were selected as pivot genes.

### Consensus cluster analysis

Consensus cluster analysis was performed using the ConsensusCluster Plus package based on the expression of PRGs [22]. Only sepsis samples were retained and divided into subgroups, with a maximum subgroup classification of k = 9. The optimal grouping was selected to study the role of PRGs in sepsis. PCA analysis was conducted using the Rtsnepackage to verify the optimal grouping.

### Analysis of immune infiltration

Single sample genomic enrichment analysis (ssGSEA) was performed using GSEABase and GSVA packages [23]. This analysis provided information on the abundance of immune cells in each sample and the correlation between these significant PRGs and immune cells. Pyroptosis-related scores for each sample were calculated using the PCA algorithm to quantify the pyroptosis-related subgroups. The correlation among pyroptosis-related clusters, pyroptosis-related gene clusters, and the high and low scores of PRGs was displayed using the ggplot2 and ggallivian packages [24].

### qRT-PCR analysis, cell culture, and treatment

H9C2 cells were purchased from American Type Culture Collection (ATCC) and stimulated with 1μg/mL LPS (Sigma, USA) for 12 h to construct an *in vitro* sepsis model [25]. After treatment with LPS for 12h, total RNA was isolated and reverse-transcribed into cDNA using a kit (Thermo Fisher Scientific, USA) and analyzed by qRT-PCR with SYBR-Green PCR kit (Takara, Japan). GAPDH was used as an internal reference. The sequences are listed in S1 Table.

### Statistical analysis

Differences between groups were compared using the Kruskal-Wallis test, and the relationship between PRGs and immune cells was explored using linear regression analysis. Pyroptosis-related key genes closely related to sepsis were screened using the t-test, and the value P< 0.05 was considered statistically significant. All statistical analyses were performed using R version 4.2.0.

## Results

### Differential expression analysis of pyroptosis-related genes

**Fig 1:**
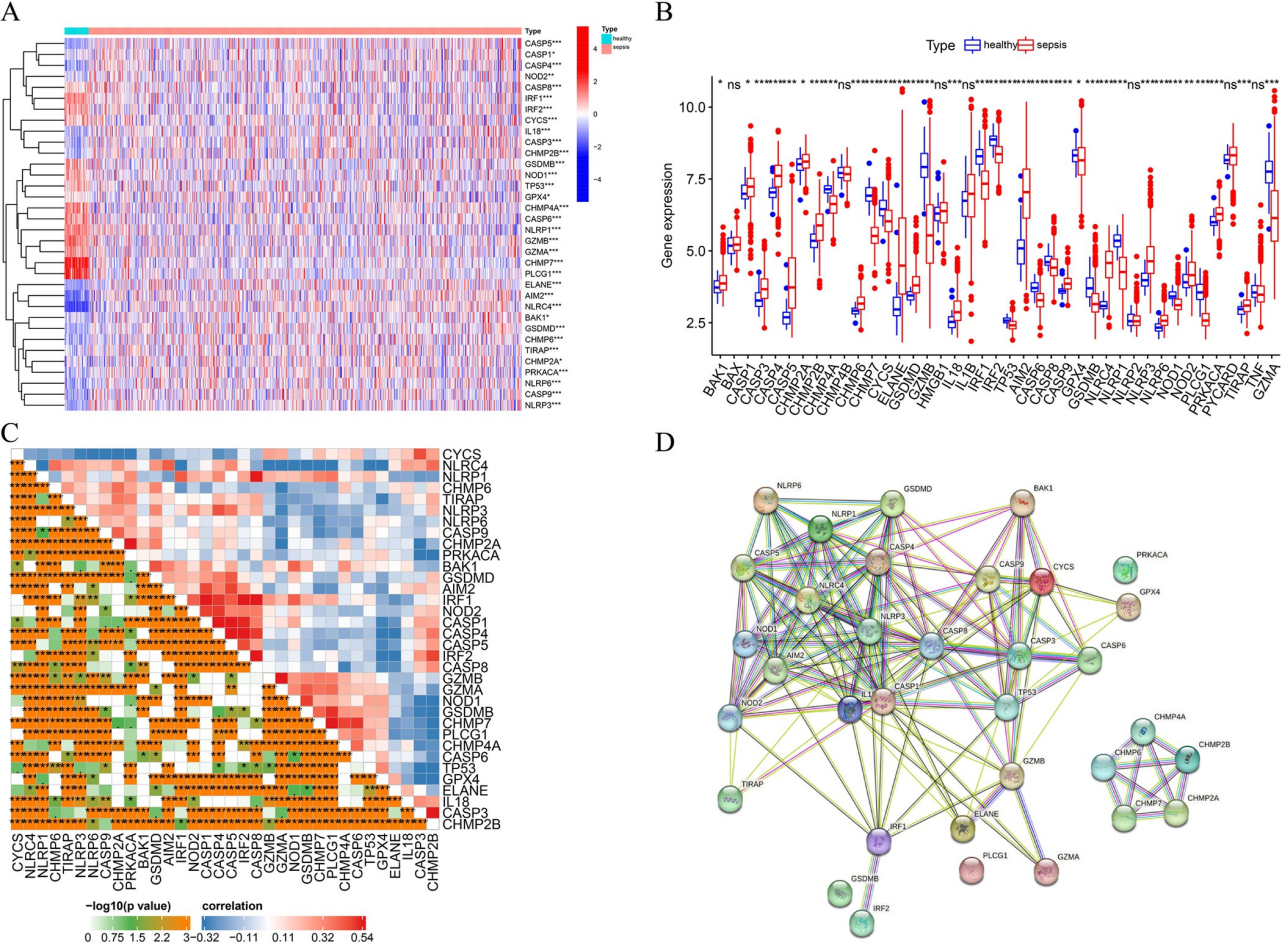
Expression level and correlation of pyroptosis-related genes in sepsis. Differential expression of pyroptosis-related genes between the sepsis group and healthy control group in heatmap (A) and boxplot (B). (C) The correlation of pyroptosis-related genes and pyroptosis-related genes. (D) The protein interaction networks of pyroptosis-related genes. ns, not significant; * *P* <0.05, **P <0.01, *** *P* <0.001.

To analyze the difference in the expression of PRGs between patients with sepsis and healthy controls, a total of 34 PRGs closely related to sepsis were identified **(Fig 1A)**. Comparative analysis revealed sepsis upregulation of BAK1, CASP1, CASP3, CASP4, CASP5, CHMP2A, CHMP2B, CHMP6, ELANE, GSDMD, IL18, AIM2, CASP9, NLRC4, NLRP3, NLRP6, NOD2, PRKACA, TIRAP, while downregulation of CHMP4A, CHMP7, CYCS, IRF1, IRF2, TP53, CASP6, CASP8, GPX4, GSDMB, NLRP1, NOD1, PLCG1, and GZMA was observed in patients with sepsis compared to the healthy control group **(Fig 1B)**. The correlation among PRGs was also revealed, as depicted in **(Fig 1C)**. The differentially expressed genes (DEGs) were used as input for the STRING database to construct a protein interaction network associated explicitly with these DEGs **(Fig 1D)**.

### Model construction and selection

**Fig 2:**
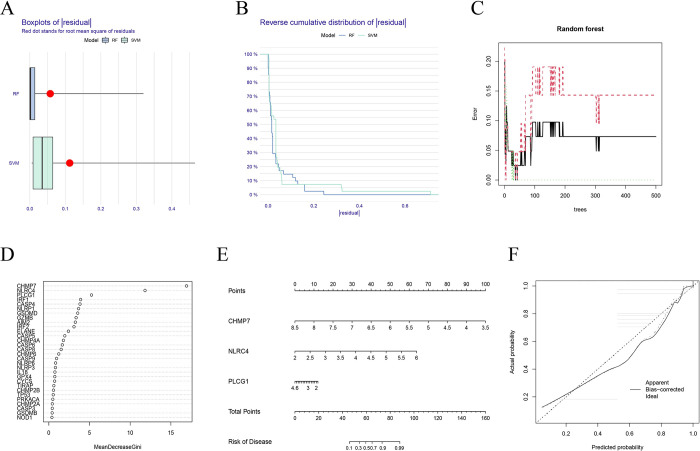
Construction of random forest (RF) model and Support Vector Machine (SVM) model. (A) The residual box plot shows the residual distribution of RF and SVM models, and a lower residual value indicates that the model is more meaningful and accurate. (B) The residual reverse cumulative distribution map shows the residual distribution of RF and SVM models. (C) The number of decision trees on the error rate. (D) The importance of the 30 differential expression genes in RF model. (E) The predictive nomogram is constructed based on 7 pyroptosis-related genes. (F) The construction of the calibration curve for the nomogram, the closer the distance between the solid and dashed lines, the higher the accuracy of the model.

To predict the occurrence of sepsis, a RF and SVM model was constructed from 34 differentially expressed PRGs. The residual box graph **(Fig 2A)** and the residual inverse cumulative distribution graph **(Fig 2B)** showed that the RF model had the smallest residual, indicating its suitability for predicting the occurrence of sepsis. Therefore, the RF model was selected as the best model for this study. When establishing an RF training model with over 200 trees, a reduction in the variation range of the model error rate was observed, indicating a tendency toward stabilization **(Fig 2C)**. The importance score map helped to identify PRGs with scores higher than five, which were then selected for further analysis **(Fig 2D)**. A nomograph was constructed based on the expression level of PRGs with gene scores above five, specifically CHMP7, NLRC4, and PLCG1, which were identified as feature biomarkers **(Fig 2E)**. The scores of each gene in sepsis were obtained, and the scores of these characteristic PRGs were summed up. Based on total scores, the prevalence of sepsis was predicted. The calibration curve showed that the positive rate of diagnosis of sepsis using this nomogram model was consistent with the actual positive rate **(Fig 2F)**. To further validate the diagnostic effectiveness of these three feature biomarkers, receiver operating characteristic (ROC) analysis suggested that the area under the curve for CHMP7, NLRC4, and PLCG1 was 0.986, 0.974, and 0.955, respectively **(S1 Fig)**. The results of the ROC analysis illustrated the satisfactory diagnostic ability of CHMP7, NLRC4, and PLCG1 for sepsis. In addition, the expression of CHMP7, NLRC4, and PLCG1 in an *in vitro* sepsis model was further validated. The qRT-PCR results showed that the expression of CHMP7 was downregulated, while NLRC4 and PLCG1 expression was upregulated in H9C2 cells stimulated with LPS to mimic *in vitro* sepsis model **(S2 Fig)**. These results confirmed the diagnostic effectiveness and differential expression of the three feature biomarkers in sepsis.

### Weighted gene co-expression network analysis (WGCNA)

**Fig 3:**
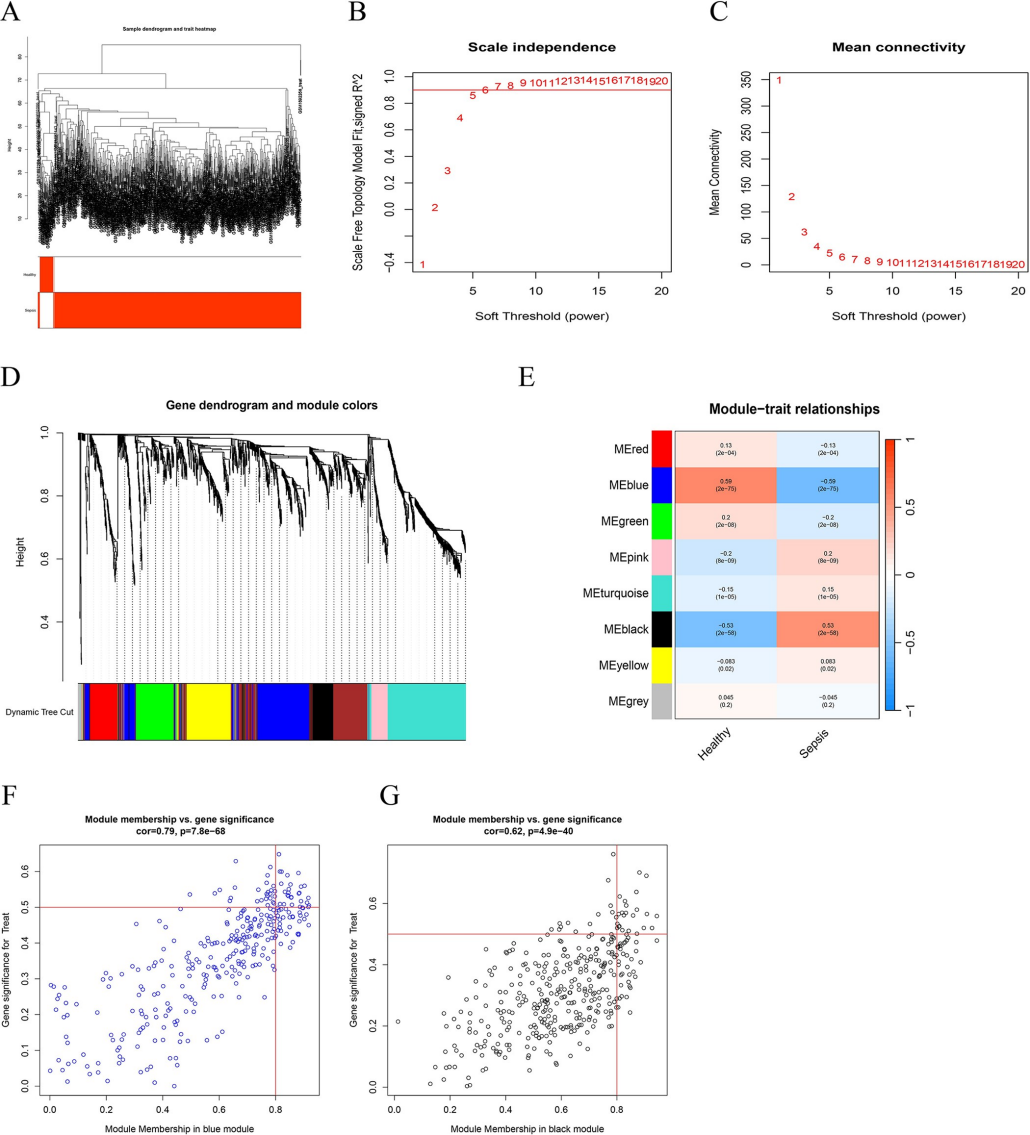
Construction of weighted gene co-expression network analysis. (A) The sample cluster tree diagram based on Euclidean distance. (B-C) Determination of soft threshold parameters. (D) Dendrogram of pyroptosis-related genes based on phase dissimilarity measure clustering. (E) Heatmap of correlation between module feature genes and disease. Scatterplot of gene significance in yellow module (F) and black module (G).

To bring new understanding to the pathogenesis, clinical diagnosis, and treatment of sepsis, WGCNA was used to screen gene modules and pivotal genes related to sepsis. A total of 5000 genes with the highest average expression were selected to construct a gene co-expression module. After processing the dataset, outlier detection was performed, and the analysis results showed no significant outliers were present **(Fig 3A)**. Consequently, the gene clustering module and clinical grouping phenotype were analyzed. Determining the soft thresholding power was necessary for constructing a co-expression network using WGCNA. In this study, the values ranging from 1 to 20 were tested, and calculated the scale-free topology fitting index R^2^ and mean connectivity for each soft threshold were **(Fig 3B and 3C)**. The R^2^ corresponded to the soft threshold v 0.8, and the average connectivity was close to zero, indicating that the network met the scale-free condition. Therefore a soft threshold value β = 8 was selected for further studies. A hierarchical clustering tree was constructed using a dynamic hybrid, where each leaf on the tree represented a gene. Genes with similar expression data clustered together to form branches representing gene modules. This process generates eight modules **(Fig 3D)**. The correlation between gene expression and different disease groups was calculated for each module. Among the modules identified, the blue and black modules showed the most significant correlation with sepsis, with a correlation coefficient of 0.59 and -0.53, respectively (P<0.05). Notably, the blue module exhibited the most positive correlation with sepsis and healthy groups, indicating its critical role in sepsis **(Fig 3E)**. Additionally, the correlation between module membership (MM) and gene significance (GS) within the yellow and black modules revealed a significant linear correlation **(Fig 3F and 3G).**

### Identification of pyroptosis-related clusters in sepsis

**Fig 4:**
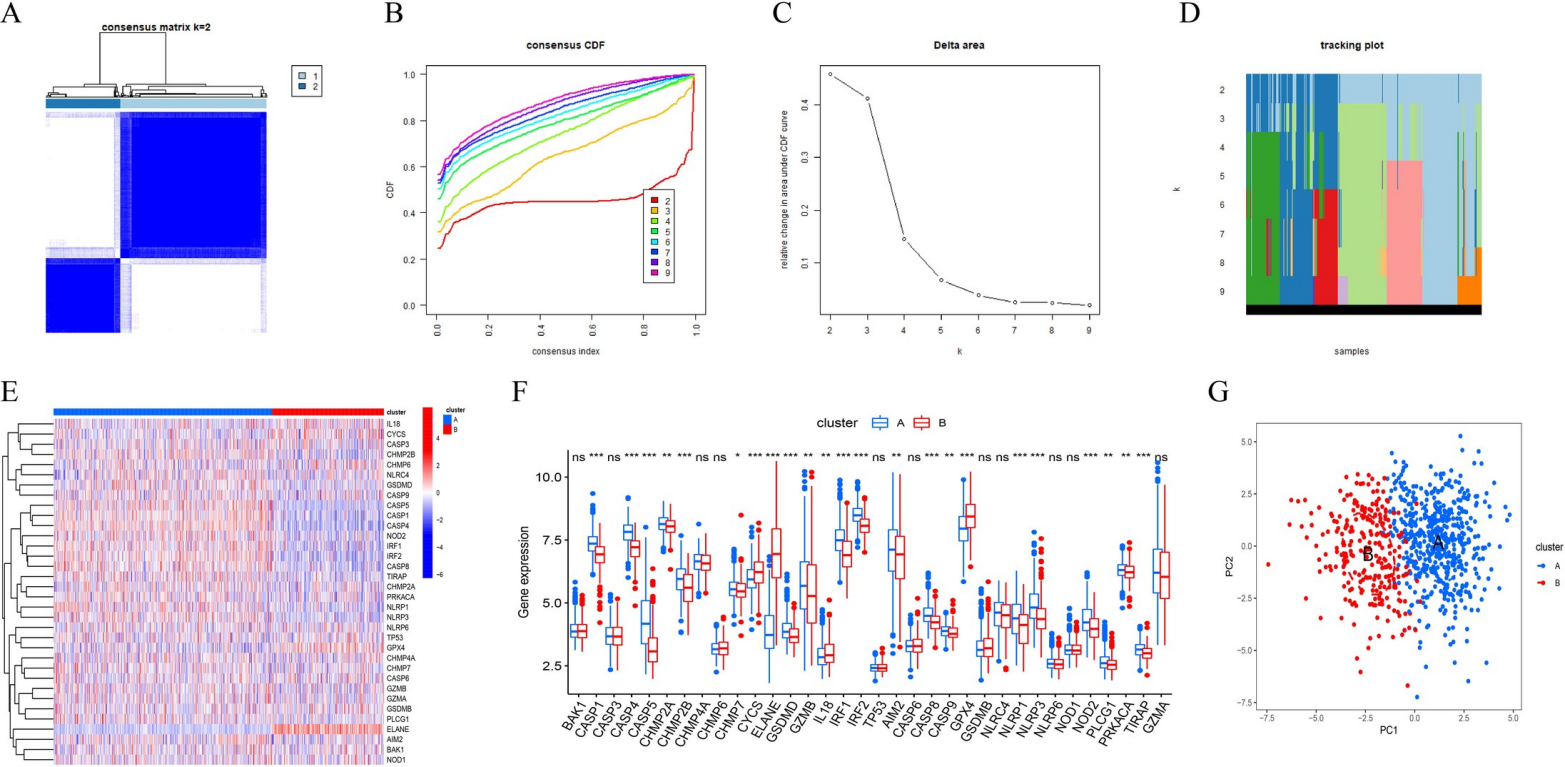
Consensus clustering analysis. (A) Two different subtypes of sepsis are distinguished. The cumulative distribution function (CDF) curve (B), delta area graph (C) and tracking plot (D) is used to decided the optimal value. (E-F) Differential expression of pyroptosis-related genes between the cluster A and B. (G) PCA analysis shows significant differences between different clusters. ns, not significant; * *P* <0.05, **P <0.01, *** *P* <0.001.

A consistent clustering algorithm was performed to identify the different subtypes of sepsis samples that were classified based on the expression level of PRGs. The optimal value of k, representing the number of clusters, was determined using the results of the cumulative distribution function (CDF) curve, delta area graph, and tracking plot. The clustering effect was best when k = 2 (**Fig 4A–4D**). All sepsis patients were divided into two sets (clusters A and B), and differential expression analysis of PRGs was conducted between the two clusters (**Fig 4E and 4F**). Principal component analysis (PCA) further confirmed significant differences in the distribution of the two clusters (**Fig 4G**).

**Fig 5:**
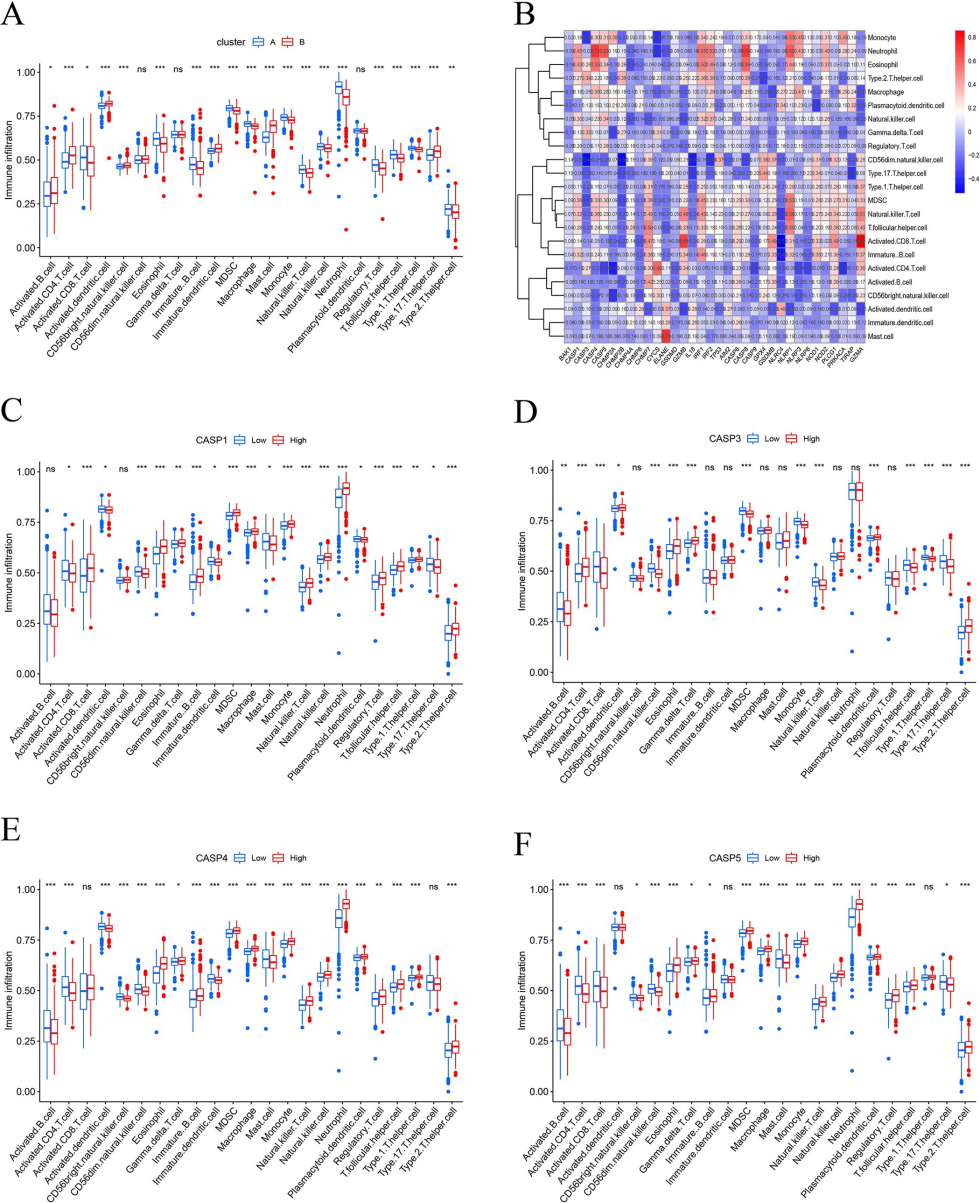
Immune cells analysis. (A) The ssGSEA analysis show a significant differents in the infiltration of immune cells between two clusters. (B) The correlation of immune cells and pyroptosis-related genes. (C-F) The difference of immune cells in sepsis patients with higher and lower expression of CASP1, CASP3, CASP4, CASP5. ns, not significant; * *P* <0.05, **P <0.01, *** *P* <0.001.

To investigate the role of PRGs in immune cells, the association between the two clusters and immune cells was evaluated separately using the single-sample gene set enrichment analysis (ssGSEA) algorithm. A significant difference was observed in the infiltration of most immune cells between the two groups (**Fig 5A**). The results of ssGSEA also confirmed the correlation between GRGs and immune cells in sepsis (**Fig 5B**). Moreover, differences in the expression of CASP1, CASP3, CASP4, and CASP5 could help distinguish the level of immune cell infiltration in sepsis (**Fig 5C–5F**).

### Identification of pyroptosis-related gene clusters in sepsis

**Fig 6:**
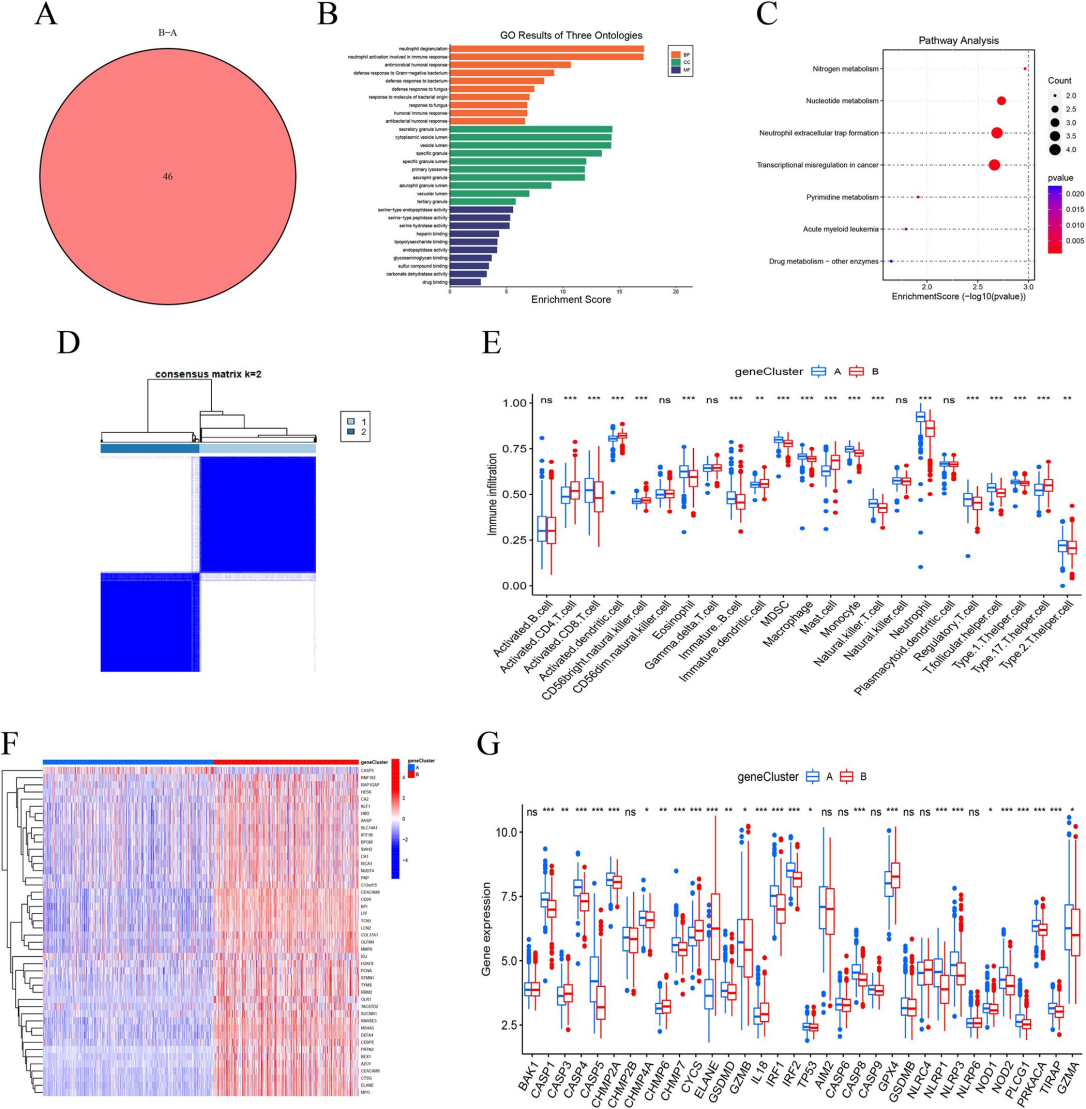
The consensus clustering analysis of gene clusters. (A) The Venn plot show the numbers of differentially expressed genes between cluster A and B. (B-C) The functional enrichment analysis of differentially expressed genes between cluster A and B. (D) Two different pyroptosis-related gene clusters are distinguished. (E) The difference of immune cells between gene cluster A and B. (F-G) The heatmap and boxplot show the difference of pyroptosis-related genes in two gene clusters. ns, not significant; * *P* <0.05, **P <0.01, *** *P* <0.001.

To further investigate the potential biological role of pyroptosis-related clusters, 46 DEGs between clusters A and B was identified (**Fig 6A**). Functional enrichment analysis of these DEGs indicated primarily involvement in immune and metabolism processes (**Fig 6B and 6C**). The consensus clustering algorithm also classified sepsis patients into two pyroptosis-related gene clusters (**Fig 6D**). The ssGSEA analysis showed that these pyroptosis-related gene clusters effectively distinguish the infiltration of immune cells (**Fig 6E**). Heat maps and boxplot results display significant differences in the expression of most PRGs between the two pyroptosis-related gene clusters (**Fig 6F and 6G**).

### Relation of pyroptosis-related subgroups and cytokines

**Fig 7:**
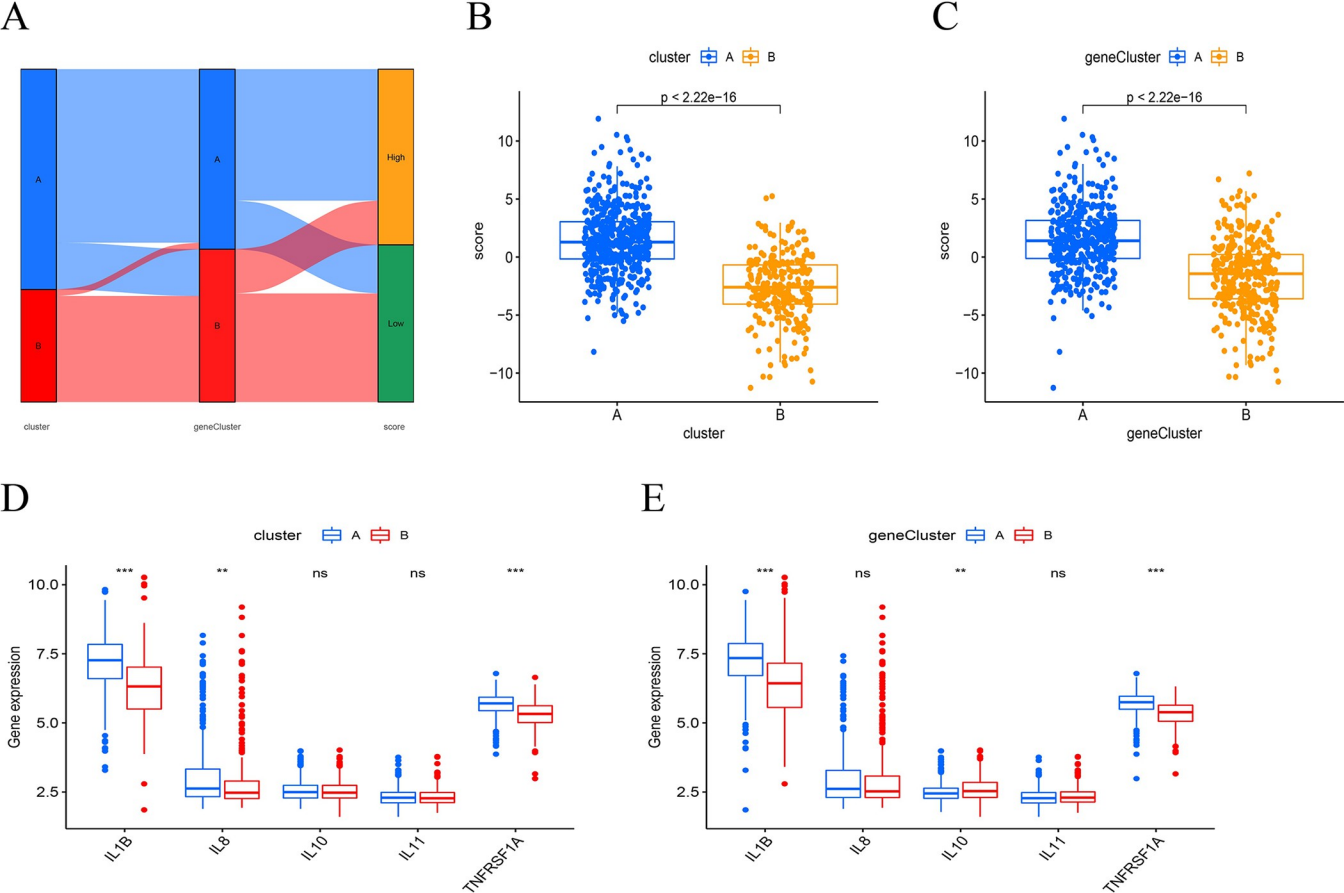
The role of pyroptosis-related genes in sepsis. (A)The Sankey plot reveals relationship between pyroptosis-related clusters, pyroptosis-related gene clusters, and pyroptosis-related score groups. (B-C) The pyroptosis-related score in different pyroptosis-related clusters and pyroptosis-related gene clusters. (D-E) The expression level of IL-1B, IL-8, IL-10, IL-11 and TNFRSF1A in different pyroptosis-related clusters and pyroptosis-related gene clusters. ns, not significant, **P <0.01, *** *P* <0.001.

To estimate the risk for each patient with sepsis, a pyroptosis-related score was calculated using a PCA algorithm based on the expression levels of PRGs. A Sankey diagram revealed the correlation between pyroptosis-related subgroups and pyroptosis-related scores (**Fig 7A**). Furthermore, the pyroptosis-related score was significantly higher in pyroptosis-related cluster A and gene cluster A (**Fig 7B and 7C**). Moreover, the correlation between apoptosis-related subgroups and cytokines was analyzed. The expression levels of interleukin (IL)-1B and TNF receptor superfamily member 1A (TNFRSF1A) were upregulated in cluster A and gene cluster A, while IL-8 expression was upregulated in cluster A but not in the gene cluster, and IL-10 expression was downregulated in gene cluster A but not in cluster A. No significant difference was observed in the expression of IL-11 (**Fig 7D and 7E**). These results indicated a significant relationship between cluster and gene cluster about inflammation.

## Discussion

Sepsis is a life-threatening condition characterized by a systemic inflammatory response caused by infection or known factors, leading to septic shock and multiple-organ dysfunction. The pathophysiological mechanisms of sepsis have yet to be fully elucidated, although the sepsis consensus has been revised three times [4,26,27]. Therefore, investigating the potential mechanisms and diagnostic biomarkers of sepsis from the machine learning perspective is significant in finding treatment targets for sepsis and improving patient symptoms and prognosis.

With the development, popularization, and application of high-throughput sequencing technology, transcriptome, and other information can be obtained in the pathological process of many diseases [28]. These extensive data can greatly assist researchers in exploring disease occurrence, development, and prognosis mechanisms and patterns. Analyzing and mining transcriptome data through machine learning techniques, finding key disease markers, and establishing prediction models are significant for clinical diagnosis, treatment, and prognosis evaluation. Pyroptosis is a recently detected form of inflammatory programmed cell death, characterized by cell swelling, rupture, dissolution, and release of proinflammatory factors, such as interleukin (IL)-1β and IL-18 [29]. However, there are limited studies on the mechanism of pyroptosis in sepsis. This study conducted differential expression analysis between sepsis and healthy control samples and employed RF and SVM models to predict the prevalence of sepsis. Based on the performance of the RF model, a predictive nomogram model was constructed, and the unique clinical diagnostic advantages of the RF model were verified. The study identified seven important PRGs including CHMP7, NLRC4, PLCG1, IRF1, CASP4, NLRP1, GSDMD associated with sepsis.

The NOD-like receptor family, pyrin domain-containing protein 4 (NLRC4) inflammasome, belongs to the innate immune system and triggers the release of IL-1β and IL-18, leading to inflammatory reactions [30]. Studies have found an immune interaction between autophagy and inflammasome in sepsis [31]. Additionally, NLRC4 has been identified as a diagnostic marker for pediatric sepsis [32]. During sepsis, NLRC4 activation contributes to pathological and physiological changes, leading to multiple-organ dysfunction. Subsequent studies have revealed that similar to GSDMD, other members of the GSDM family can also mediate cell membrane rupture and pyroptosis, further emphasizing their role in this process [33,34]. Numerous studies have shown that the factors involved in the NLPR3/Caspase-1/GSDMD process, a classical pathway associated with pyroptosis, are highly expressed in patients and animals with sepsis. However, blocking these factors or knocking out specific genes in this pathway has been found to reduce the systemic inflammatory response, providing some alleviation in organ function damage [35,36].

Clinical observation studies have indicated that patients with sepsis exhibit a decrease in peripheral blood CD3+T, CD4+T, and CD4+/CD8+T cell ratios, which are related to the severity of the disease [37]. Moreover, the number of Th17 cells in the peripheral blood of patients with sepsis complicated by traumatic hemorrhagic shock significantly decreases [38]. In the early stage of septic shock, there is a considerable reduction in the number of circulating CD4+CD25+T cells and CD4+CD25-T cells, followed by a rapid increase in Treg cells and sustained low levels of CD4+CD25-T cells [39]. Therefore, the consensus clustering method was employed to identify two pyroptosis-related patterns (cluster A and cluster B) based on the expression levels of PRGs.Both classification patterns effectively distinguish between two distinct subtypes of patients with sepsis. Cluster B shows a high correlation with an imbalance in the Th1/Th2 cell ratio, a higher proportion of Th17 cells, and lower expression levels of IL-1β, IL-8, and TNFRSF1A. This study explores the relationship between pyroptosis-related patterns, sepsis, and inflammatory factors and provides insights into potential directions for future clinical diagnosis and treatment of sepsis.

However, certain limitations are there in this study. Firstly, despite quality control, homogenization, and standardization of the original data, there is still a possibility of bias in the results due to the small sample size, and a single blood sample may need broader representativeness. Secondly, although PRGs related to sepsis have been identified, further experimental validation is required to elucidate their function and mechanism in sepsis. In addition, these conclusions were reached by working with a single dataset in a public database, lacking multiple clinical data to validate the relationship between pyroptosis and sepsis.

## Conclusion

In conclusion, this study established a novel risk prediction model based on PRGs that better predicts the risk of sepsis. Additionally, it identified two distinct subtypes of patients with sepsis based on pyroptosis-related clusters (cluster A and cluster B). Cluster B is strongly associated with a lower proportion of Th17 cells and has lower expression levels of inflammatory factors. The finding of this investigation provides specific reference values for future basic research on the immune aspect of pyroptosis in sepsis.

## Supporting information

S1 Table Primer sequence of genes in qRT-PCR. (DOCX)

S1 Fig Receiver Operating Characteristic (ROC) analysis. ROC curve of three feature biomarkers including CHMP7 (A), NLRC4 (B), PLCG1 (C).
(TIF)

S2 Fig The results of qRT-PCR analysis. The expression of CHMP7 (A), NLRC4 (B), PLCG1 (C) were detected by qRT-PCR in an *in vitro* sepsis model.
(TIF)
