# Supplementary material for: The role of pyroptosis-related genes in the diagnosis and subclassification of sepsis
Source: PLoS One. 2023 Nov 8;18(11):e0293537. doi: 10.1371/journal.pone.0293537 (PMC10631697; doi:10.1371/journal.pone.0293537)
Supplement: S1 Table — (DOCX) [file pone.0293537.s001.docx]

Primer sequence of genes in qRT-PCR.

|  | Forward sequence | Reverse sequence |
| --- | --- | --- |
| CHMP7 | AAGCCTCTCAAGTGGACTCTT | ACAGACGATACACCTCCTCAG |
| NLRC4 | TCAGAAGGAGACTTGGACGAT | GGAGGCCATTCAGGGTCAG |
| PLCG1 | GGAAGACCTCACGGGACTTTG | GCGTTTTCAGGCGAAATTCCA |
| GAPDH | GGAGCGAGATCCCTCCAAAAT | GGCTGTTGTCATACTTCTCATGG |
