# Supplementary figures and images for: The role of pyroptosis-related genes in the diagnosis and subclassification of sepsis
Source: PLoS One. 2023 Nov 8;18(11):e0293537. doi: 10.1371/journal.pone.0293537 (PMC10631697; doi:10.1371/journal.pone.0293537)

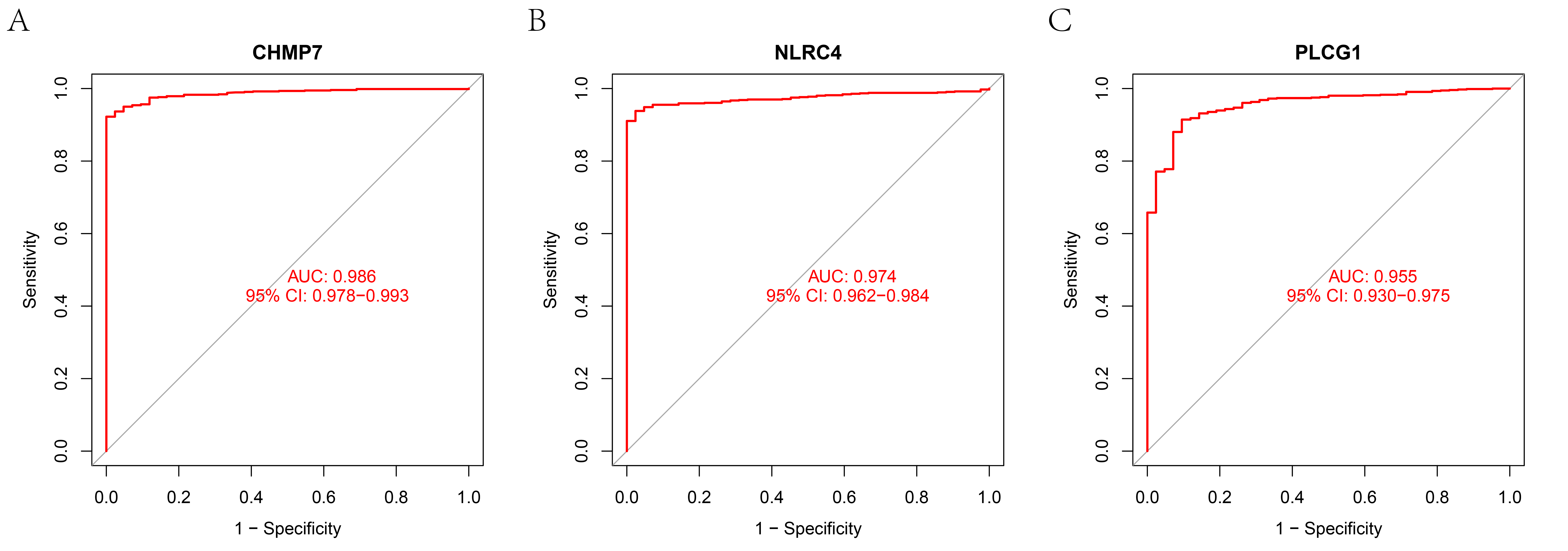

Supplement: S1 Fig — ROC curve of three feature biomarkers including CHMP7 (A), NLRC4 (B), PLCG1 (C). (TIF) [file pone.0293537.s002.tif]

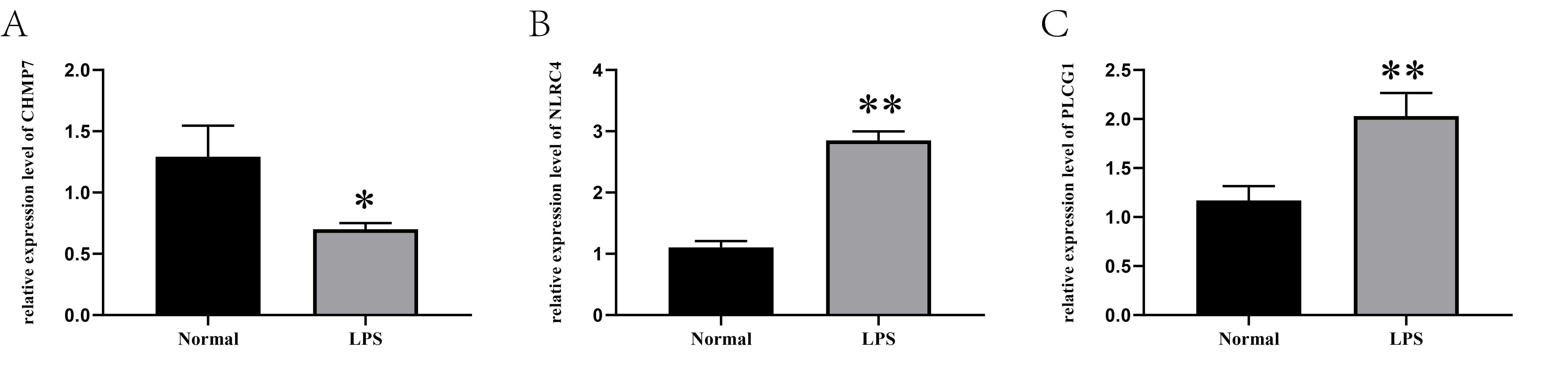

Supplement: S2 Fig — The expression of CHMP7 (A), NLRC4 (B), PLCG1 (C) were detected by qRT-PCR in an in vitro sepsis model. (TIF) [file pone.0293537.s003.tif]
